# Association of antithyroglobulin antibody with iodine nutrition and thyroid dysfunction in Nepalese children

**DOI:** 10.1186/s13044-019-0067-z

**Published:** 2019-07-09

**Authors:** Binaya Tamang, Saroj Khatiwada, Basanta Gelal, Shrijana Shrestha, Kishun Deo Mehta, Nirmal Baral, Gauri Shankar Shah, Madhab Lamsal

**Affiliations:** 1 Department of Biochemistry, Universal College of Medical Sciences, Bhairahawa, Nepal; 2 School of Medical Sciences, UNSW SYDNEY, Sydney, Australia; 3 Department of Biochemistry, B P Koirala Institute of Health Sciences, Ghopa, Dharan, Nepal; 4 Department of Paediatrics and Adolescent medicine, B P Koirala Institute of Health Sciences, Dharan, Nepal

**Keywords:** Antithyroglobulin antibody, Iodine deficiency, Nepal, School children, Thyroid autoimmunity, Thyroid dysfunction

## Abstract

**Background:**

Aberrant iodine intake and thyroid autoimmunity affect thyroid function. Deficiencies of iodine including thyroid disorders have serious impact on child physical and mental development. This study was conducted to investigate iodine nutrition, thyroid function and thyroid autoimmunity in the Nepalese children, and explore the association of thyroidal autoimmunity with iodine nutrition and thyroid dysfunction.

**Methods:**

Five schools from Udayapur district of eastern Nepal were selected for the study. A total of 213 school children aged 6–12 years were enrolled, and anthropometric data, urine samples and blood samples were collected. Urinary iodine concentration (UIC), free triiodothyronine (fT3), free thyroxine (fT4), thyroid stimulating hormone (TSH), and antithyroglobulin antibody (TgAb) was measured. Independent T test, Man-Whitney test, Chi-square test and Fisher’s Exact test were used for testing statistical significance. Spearman’s correlation analysis was done to find association between variables.

**Results:**

The median UIC with IQR, mean ± SD fT3, mean ± SD fT4, median TSH and TgAb with IQR was 150.0 μg/L (102.8; 204.0), 2.49 ± 0.83 pg/ml, 1.33 ± 0.42 ng/dl, 2.49 mIU/L (1.58; 4.29), and 21.40 IU/ml (15.54; 31.20) respectively. Elvated TgAb (≥30 IU/ml, thyroid autoimmune condition) was seen in 25.8% (*n* = 55) children. UIC was less than 100 μg/L in 17.4% (*n* = 37) of the children. Subclinical hypothyroidism, overt hypothyroidism and sublinical hyperthyroidism was seen in 1.4% (n = 3), 3.3% (*n* = 7) and 3.8% (*n* = 8) children respectively. A strong association of TgAb with UIC (r = − 0.210, *p* = 0.002) and thyroid hormones; fT3 (r = − 0.160, *p* = 0.019), fT4 (r = − 0.275, *p* < 0.001), and TSH (r = 0.296, *p* < 0.001) was seen. The relative risk for thyroid autoimmunity in children with UIC less than 100 μg/L was 1.784 (95% CI: 1.108–2.871, *p* = 0.024). Similarly, children with thyroid autoimmunity had higher relative risk [7.469 (95% CI: 2.790–19.995, *p* < 0.001)] for thyroid dysfunction.

**Conclusions:**

School children of eastern Nepal have adequate iodine nutrition. Thyroid autoimmunity is very common, while thyroid dysfunction is sparse in children. An association of thyroid autoimmunity with iodine nutrition and thyroid dysfunction was seen in children.

## Background

Thyroid gland produces hormones essential for normal growth and development of the body. Deficiency of thyroid hormones, a condition called as hypothyroidism, or excess of thyroid hormones called as hyperthyroidism, both alter normal body physiology and metabolism [1, 2]. The prevalence of thyroid dysfunction ranges from 1 to 10% among adults from general population, however, prevalence varies across different groups [3]. In Nepal, high prevalence of thyroid disorders has been reported among patients visiting tertiary hospitals, and those with chronic diseases [4–7]. Several studies have shown thyroid dysfunction to be common among pregnant women and school children of eastern Nepal [8–10].

The most common cause of thyroid disorders worldwide is iodine deficiency except in the areas with adequate iodine nutrition where thyroid autoimmunity is the major cause [11]. Severe iodine deficiency causes hypothyroidism, while at the same time excessive iodine intake can also negatively impact thyroid function [12]. In addition, the development of autoantibodies against thyroid proteins mainly thyroid peroxidase (TPO), thyroglobulin (Tg), and thyroid stimulating hormone (TSH) receptor can lead to thyroid disorders [13]. Aberrant iodine intake can play a crucial role in the development of thyroid autoimmunity, and affect the rate of thyroid diseases [14]. There is less information regarding thyroid autoimmunity in Nepalese children.

In the past, iodine deficiency used to be a significant health problem in Nepal, but ever since universal salt iodization program (USI) was initiated in 1993, iodine deficiency disorders have progressively dropped [15]. Our studies among the school children of eastern Nepal show good improvement in iodine nutrition as indicated by rising median urinary iodine concentration (UIC) of the population over the past years, which is consistent with the increasing usage of adequately iodized salt by the households [16–19]. Because of the risk associated with excess or inadequate iodine intake, it is important to monitor iodine intake and iodine nutrition in the population.

Our previous studies were focused on iodine nutrition, iron status, thyroid function and their relationships in the children [9, 10, 17]. In this study, we aimed to evaluate iodine nutrition, thyroid function and thyroid autoimmunity including their relationship in the school children from eastern Nepal.

## Methods

This cross-sectional study was conducted by the Department of Biochemistry in collaboration with department of Pediatrics and Adolescent Medicine of B. P. Koirala Institute of Health Sciences (BPKIHS), Dharan, Nepal in the Udayapur district of eastern Nepal in the year 2015–2016. Udayapur district has a mixed geography comprising plain (low altitude) and hilly (high altitude) areas. Three village development committee (VDC); Katunjebawala, Chaudandi and Siddhipur were selected for the current study. One VDC (Katunjebawala) lies at low altitude of around 306 m above the sea level whereas the other two (Chaudandi and Siddhipur) lie at high altitude, around 2300 m above the sea level. Those VDCs were selected randomly as a representative of low and high altitude regions of Udayapur district. A total of five schools (two from Katunjebawala, one from Chaudandi and two from Siddhipur) were chosen for the subject recruitment. From each school, children aged 6–12 years were enrolled. Prior to the enrollment, the objectives and benefits of the study were explained to the school authorities including guardians and participants of the study. Consent to participate in the study was taken from each child and their guardian. Participants taking micronutrient supplements, with severe illness and those not willing to participate were excluded from the study. The ethical clearance for this study was provided by the Institutional Review Committee (IRC) of BPKIHS.

Two hundred thirteen children participated in the study. The sample size was estimated based on assumption of approximate prevalence of iodine deficiency (ID) (15%), thyroid dysfunction (20%), and thyroid autoimmunity (15%) in the Nepalese population. From each subject anthropometric data (height, weight) was recorded, and then urine and blood samples were collected at the same time. Casual urine samples were collected in 10 ml sterile tubes and blood samples (2 ml) in plain vials. Serum was separated within an hour of blood collection. Urine and serum samples were transported to the biochemistry laboratory of BPKIHS maintaining cold chain and stored at -20^o^ C until analyte measurement. UIC was estimated in the urine samples using ammonium persulfate digestion microplate (APDM) method (based on Sandell-Kolthoff reaction), in a specially designed apparatus, sealing cassette [20]. In the serum, free tri-iodothyronine (fT3), free tetra-iodothyronine (fT4), thyroid stimulating hormone (TSH) and antithyroglobulin antibody (TgAb) were measured. Thyroid hormones; fT3, fT4 and TSH were measured by ELISA method using commercial kits from Diametra Company. TgAb was estimated by chemiluminescence immunoassay (CLIA) method using MAGLUMI 1000 CLIA kits.

The reference ranges provided by the kits manufacturer for fT3, fT4, TSH, and TgAb were 1.4–4.2 pg/ml, 0.8–2.2 ng/dL, 0.39–6.16 mIU/L, and < 30 IU/ml respectively. Using above reference ranges, children were classified for thyroid autoimmunity (thyroid autoimmunity if TgAb≥30 IU/ml, no thyroid autoimmunity if TgAb< 30 IU/ml) and thyroid function status (euthyroid, overt and subclinical hypothyroid, and subclinical hyperthyroid). Iodine status was classified as insufficient (UIC < 100 μg/L) or sufficient (UIC ≥ 100 μg/L) iodine nutrition based on UIC cutoff 100 μg/L, and as severe ID, moderate ID, mild ID, sufficient and excessive iodine nutrition based on WHO criteria [21, 22].

The data was entered in MS excel and analyzed by SPSS version 20.2 software after checking for the normality using Shapiro-Wilk test. The data were expressed as mean ± SD or median with interquartile range (IQR). Independent T test, Man-Whitney test, Chi-square test and Fisher’s Exact test were used for testing statistical significance at 95% confidence interval. For measuring the association among variables, Spearman’s correlation analysis was done. The relative risk for thyroid autoimmunity in children with insufficient UIC as compared to those with sufficient UIC, relative risk for thyroid dysfunction in children with insufficient UIC and thyroidal autoimmunity as compared to those with sufficient UIC and non-thyroidal immunity respectively was calculated at 95% confidence interval. A *p* value< 0.05 was considered statistically significant.

## Results

### Anthropometric measurements

The data were collected from 213 children, 66 were from low altitude (VDC: Katunjebawala) and 115 from high altitude (VDCs: Chaudandi and Siddhipur). The mean ± SD age, height and weight of the children were 10.2 ± 1.7 years, 125.9 ± 9.5 cm and 25.4 ± 5.7 kg respectively. The mean ± SD age, height and weight in the children from low altitude were 10.5 ± 1.6 years, 128.0 ± 8.4 cm and 26.8 ± 4.6 kg respectively. Among the children from high altitude, mean age, height and weight was 10.1 ± 1.8 years, 124.9 ± 9.9 cm and 24.8 ± 6.0 kg respectively. The number of males and females in the study was 115 and 98 respectively. The mean ± SD age, height and weight was 10.4 ± 1.7 years, 126.2 ± 10.0 cm and 25.3 ± 5.3 kg respectively in boys. In girls, the mean ± SD age, height and weight was 10.1 ± 1.7 years, 125.5 ± 8.9 cm and 25.5 ± 6.1 kg respectively.

### UIC, thyroid hormones and anti-Tg antibodies levels

As shown in Table 1, median UIC with IQR, mean ± SD fT3, mean ± SD fT4, median TSH and TgAb with IQR were 150.0 μg/L (102.8; 204.0), 2.49 ± 0.83 pg/ml, 1.33 ± 0.42 ng/dl, 2.49 mIU/L (1.58; 4.29), and 21.40 IU/ml (15.54; 31.20) respectively. Median UIC was significantly higher in males than females (*p* = 0.03), and in the children from high altitude than low altitude (*p* = 0.002). Median TgAb was significantly higher in the children from low altitude than from high altitude (*p* = 0.036).

**Table 1:** Median UIC, thyroid hormones and TgAb in the study population

Variables	Total *N* = 213	Sex			Altitude		
		Male *N* = 115	Female *N* = 98	*P* value	Low altitude *N* = 66	High altitude *N* = 147	*P* value
Median UIC (μg/L)	150.0 (102.8; 204.0)	166.9 (105.0; 221.0)	150.0 (105.0;192.36)	0.03	107.5 (90.8;186.5)	163.0 (108.0;212.0)	0.002
Mean fT3 (pg/ml)	2.49 ± 0.83	2.57 ± 0.79	2.41 ± 0.87	0.174	2.51 ± 0.85	2.49 ± 0.82	0.864
Mean fT4 (ng/dl)	1.33 ± 0.42	1.31 ± 0.38	1.37 ± 0.46	0.289	1.28 ± 0.43	1.36 ± 0.41	0.186
Median TSH (mIU/L)	2.49 (1.58;4.29)	2.48 (1.64;3.79)	2.56 (1.44;4.71)	0.412	2.75 (1.69; 4.77)	2.36 (1.53; 4.06)	0.222
Median TgAb (IU/ml)	21.40 (15.54;31.20)	20.60 (14.89;28.92)	21.5 (16.76;32.98)	0.217	24.65 (16.76; 41.24)	20.4 (15.3;27.98)	0.036

The data are expressed as mean ± SD or median (IQR). Independent samples T test and Mann-Whitney test was applied for testing statistical significance

### Iodine nutrition, thyroid disorders and thyroid autoimmunity

Thyroid dysfunction and thyroid autoimmunity (TgAb≥30 IU/ml) were present in 18 (8.5%) and 55 (25.8%) children respectively. About 37 (17.4%) children had UIC < 100 μg/L. Based on WHO criteria, severe, moderate, and mild ID was seen in 1 (0.5%), 8 (3.8%) and 28 (13.1%) children respectively. Iodine nutrition was excessive (UIC ≥ 300 μg/L) in 21 (9.9%) children. Insufficient iodine nutrition was found in 31.8% (*n* = 21) of the children from low altitude and 10.9% (*n* = 16) children from high altitude. Thyroid dysfunction was seen in 6.1% (*n* = 4) children residing in low altitude and 9.5% (*n* = 14) children residing in high altitude. Similarly, thyroid autoimmunity was seen in 34.8% (*n* = 23) children from low altitude and 21.8% (*n* = 32) from high altitude. Child sex and altitude did not affect thyroid function (*p* = 0.419 and *p* = 0.888 respectively). Similarly, child sex did not affect thyroid autoimmunity (*p* = 0.594) and iodine nutrition (*p* = 0.149), but altitude affected thyroid autoimmunity (*p* = 0.044) and iodine nutrition (*p* < 0.001). Type of thyroid dysfunction according to thyroid autoimmunity and iodine nutrition is shown in Table 2.

**Table 2:** Thyroid dysfunction according to autoimmunity and iodine status in the study subjects

Variables	Total N = 213	Thyroid autoimmunity			Iodine status		
		TgAb< 30 IU/ml *N* = 158	TgAb≥30 IU/ml N = 55	*P* value	UIC < 100 μg/L N = 37	UIC > 100 μg/L *N* = 176	*P* value
Euthyroidism, n (%)	195 (91.5%)	153 (71.8%)	42 (19.7%)	< 0.001	31 (14.6%)	164 (77.0%)	0.033
Overt hypothyroidism, n (%)	3 (1.4%)	–	3 (1.4%)		1 (0.5%)	2 (0.9%)	
Subclinical hypothyroidism, n (%)	7 (3.3%)	–	7 (3.3%)		4 (1.9%)	3 (1.4%)	
Subclinical hyperthyroidism, n (%)	8 (3.8%)	5 (2.4%)	3 (1.4%)		1 (0.5%)	7 (3.3%)	

The data is expressed as number (percentage). Fisher’s Exact Test was applied to test statistical significance

### Relationship of UIC with thyroid hormones and anti-Tg antibodies

**Fig. 1:**
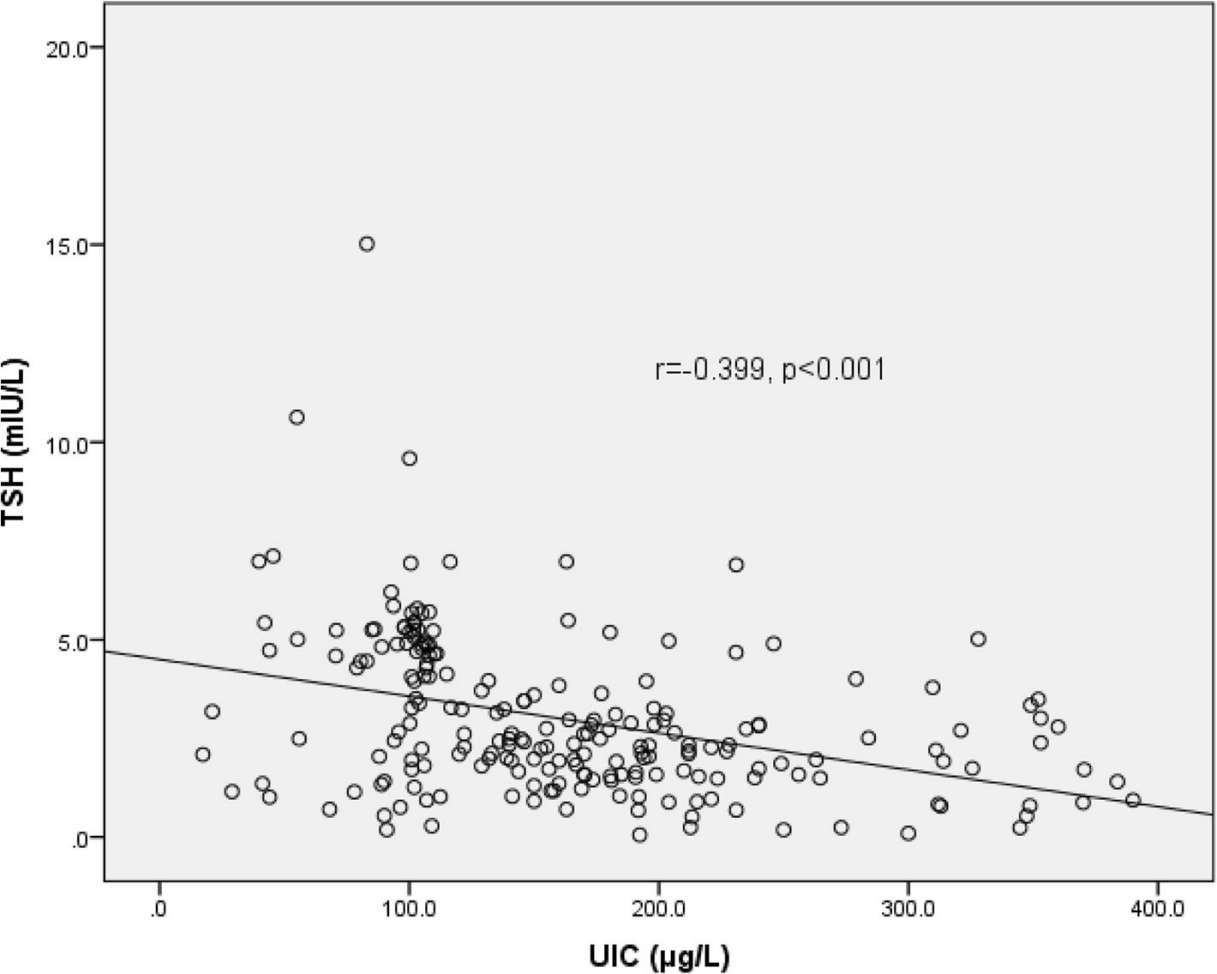
Correlation of UIC with TSH concentration

**Fig. 2:**
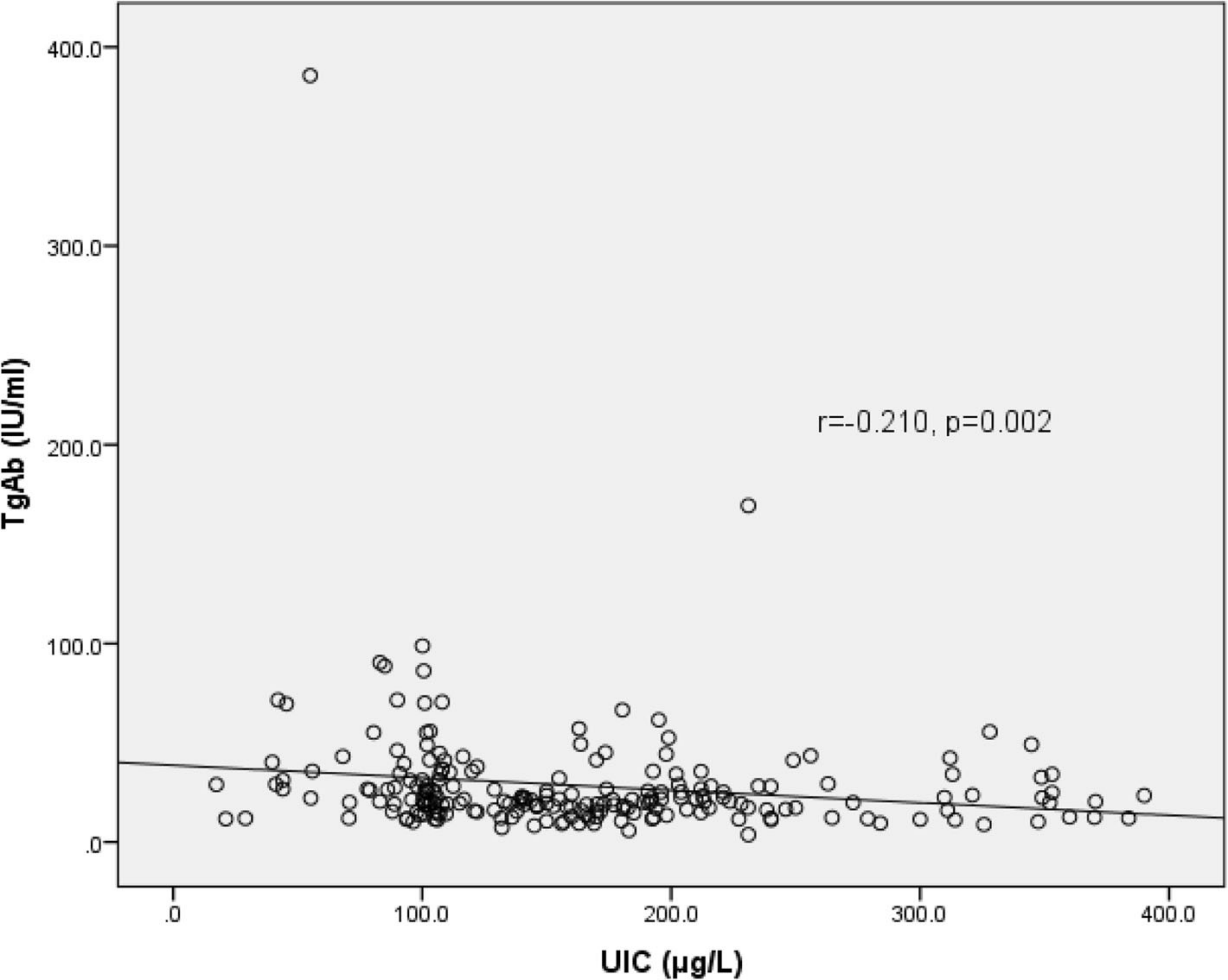
Correlation of UIC with TgAb concentration

**Fig. 3:**
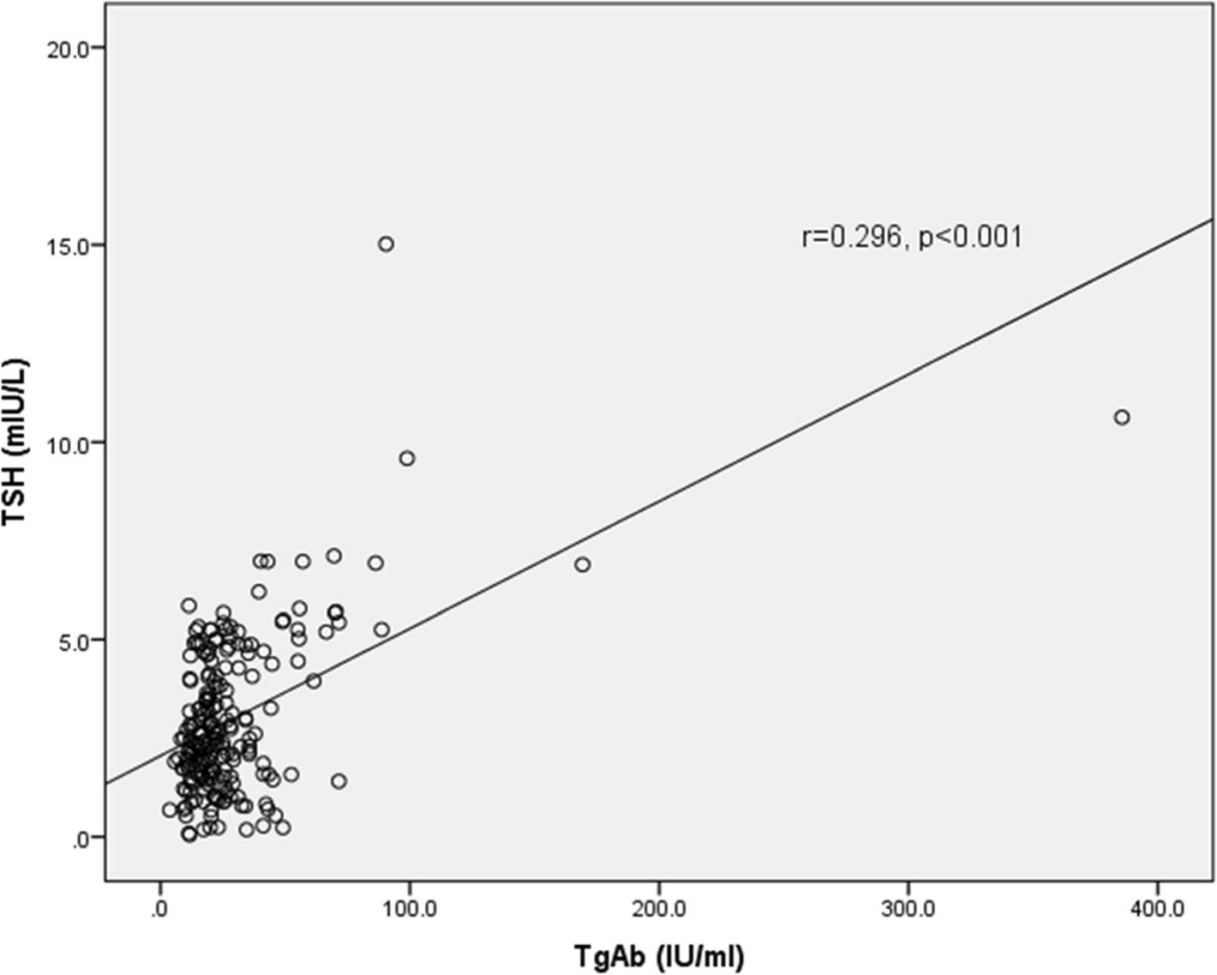
Correlation of TgAb with TSH concentration

Anthropometric measures were not associated with iodine nutrition, thyroid hormones and thyroid autoantibodies level. The correlation of UIC with TSH, UIC with TgAb, and TgAb with TSH is shown in Figs. 1, 2 and 3 respectively. A strong positive correlation of UIC with fT3 (r = 0.361, *p* < 0.001), and fT4 (r = 0.365, *p* < 0.001) was seen. UIC had negative correlation with TSH (r = − 0.399, *p* < 0.001). TgAb had significant negative correlation with fT3 (r = − 0.160, *p* = 0.019), fT4 (r = − 0.275, *p* < 0.001), UIC (r = − 0.210, *p* = 0.002), and positive with TSH (r = 0.296, p < 0.001). The relative risk of having thyroid dysfunction in children with thyroid autoimmunity was 7.469 (95% CI: 2.790–19.995, *p* < 0.001) as compared to children without thyroidal autoimmunity. Similarly, the relative risk for thyroid dysfunction in children with insufficient UIC was 2.378 (95% CI: 0.954–5.930, *p* = 0.096) as compared to children with sufficient UIC. The relative risk for thyroid autoimmunity in children with insufficient UIC was 1.784 (95% CI: 1.108–2.871, *p* = 0.024).

## Discussion

This study finds high rate of thyroid autoimmunity and a low rate of thyroid dysfunction in school children of eastern Nepal. Iodine nutrition is adequate in the population with median UIC 150 μg/L; however, 17% children had UIC < 100 μg/L. Poor iodine nutrition was found to be associated with thyroid autoimmunity and thyroid dysfunction.

Thyroid disorders are the second most common endocrine disorder worldwide [11]. In this study, thyroid dysfunction was seen in 8.5% of the children. Subclinical hyperthyroidism (3.8%) was the most common thyroid disorders followed by subclinical hypothyroidism (3.3%) and overt hypothyroidism (1.4%). Previous study by Chaudhari et al. reported subclinical hypothyroidism in 31.8 and 29.5% children of Sunsari and Dhankuta districts of eastern Nepal [23]. In another study from eastern Nepal, subclinical hypothyroidism, overt hypothyroidism and subclinical hyperthyroidism was seen in 16.3, 1.3 and 1.8% children respectively [9]. The main cause of thyroid disorders worldwide is iodine deficiency, except in the areas where iodine intake is sufficient. In such areas, autoimmunity towards thyroidal tissue is the leading cause for thyroid disorder [11].

School children are one of the most vulnerable groups for iodine deficiency. Iodine deficiency is the most common cause of mental retardation worldwide [22]. The median UIC in the present study was 150.0 μg/L μg/L, which indicates adequate iodine nutrition. The median UIC in the children from low altitude and high altitude was 107.5 μg/L and 163.0 μg/L respectively. After the launch of USI in Nepal, iodine nutrition has improved sharply [15]. Other studies by Chaudhari et al. in 2009 reported median UIC of 238.0 μg/L in Dhankuta and 294.9 μg/L in Sunsari, and Shakya et al. in 2010–2011 found median UIC of 345.6 μg/L in Tehrathum and 270.4 μg/L in Morang [10, 23]. Among the study population, 17.4% of the children had UIC < 100 μg/L, which suggest the necessity of monitoring iodine intake in such children. Previously in this region, Gelal et al., Khatiwada et al. and Shakya et al. found iodine deficiency in 22.0, 12.6, and 8.6% school children respectively [10, 17, 24]. Surprisingly, here we found higher median UIC in the children living in the high altitude than low altitude of the district. Similar trend in the median UIC was seen by Shakya et al. between the plain district (Morang) and hilly district (Tehrathum) [10]. Usually, iodine deficiency is found to be more common in the hilly and mountainous areas possibly due to the low level of iodine in the soil in such areas [25]. Our finding suggests adequate consumption of iodine rich food including iodized salts by the children living in the high altitude of the district.

For determining thyroid autoimmunity status in the children, we estimated TgAb concentration. Based on the TgAb concentration cutoff, high level of TgAb was found in 25.8% children, suggesting a high prevalence of thyroid autoimmunity. Previous data about the prevalence of thyroidal autoimmunity is not available for Nepalese children. In a study from Iran positive Tg antibodies was present in 5.3% school children [26]. In Sri Lanka, prevalence of high TgAb ranging from 14.3% (in 11 years old) to 69.7% (in 16 years old) was seen in females after salt iodization was made compulsory, pointing out the potential side effect of excess iodization [27]. In one of the study among Nepalese pregnant women, thyroid autoimmunity as estimated using anti-TPO antibodies was seen in 3.3% of the women [8]. The common antibodies developed against thyroid include TPO, TSH receptor and Tg antibodies, and such antibodies when reach a certain threshold concentration will impair thyroid function [13]. Several factors such as genetic makeup, epigenetics factor, dietary pattern, gender, environmental factors such as exposure to toxin, smoking, infection, radiation and medications, and iodine intake can trigger thyroid autoimmunity [28]. Interestingly in this study, thyroid autoimmunity was more common among children living in low altitude than living in high altitude. This might be due to the difference in iodine intake, ethnicity, genetic makeup and socio-demographic status of the children living in different altitude.

In this study, we observed a strong association of UIC with thyroid hormones namely TSH, which suggest a strong role of iodine nutrition on the thyroid function. High UIC indicates excessive iodine intake which can lead to iodine induced hyperthyroidism. After iodine supplementation, the risk for iodine induced hyperthyroidism has been reported in some African countries [29]. Our data suggest that excess iodine intake may be increasing the prevalence of subclinical hyperthyroidism in the children while at the same time contributing for hypothyroidism. Iodine is a component of thyroid hormone, so it has crucial role in maintaining normal thyroid function [12]. Both iodine deficiency and excess can coexist in the different regions of same country and contribute towards thyroid disorders [30]. Furthermore, we observed high risk for thyroid dysfunction in the children who had thyroid autoimmunity, which indicates the impact of thyroid autoimmunity on thyroid dysfunction. In the regions with adequate iodine nutrition, thyroid autoimmunity becomes a significant factor for thyroid dysfunction [11].

We observed a strong association of iodine nutrition and thyroid autoimmunity in this study. This emphasizes the importance of maintaining proper iodine nutrition in children to prevent thyroid autoimmunity development. Here we used anti-Tg antibodies to define thyroid autoimmunity instead of anti-TPO antibodies, which is one of the limitations of this study. We were interested to assess anti-Tg antibodies level in Nepalese setting as there were no previous data on this antibodies. In addition, iodine deficient children had high risk for thyroid autoimmunity than children with adequate iodine nutrition. Several studies have showed low UIC as a risk factor for thyroidal autoimmunity in the population living in iodine sufficient areas [31, 32]. At the same time, after the start of salt iodization program, several countries have reported rise in the incidence of thyroid autoimmunity possibly due to excess iodine nutrition [27, 33, 34]. A systematic review suggested that despite improvement in goiter rate through universal iodization, chronic intake of iodine through iodized salt or water increases risk for hypothyroidism in the population [35]. Thus it is important to monitor iodine intake and UIC in the population for minimizing thyroid autoimmunity and thyroid dysfunction. This study has several limitations such as small sample size that might have affected prevalence of thyroid autoimmunity including pattern of thyroid disorders, lack of anti-TPO antibodies for thyroid autoimmunity, and cross-sectional nature which cannot draw conclusion about cause and effect relationship between iodine deficiency, thyroid autoimmunity and thyroid dysfunction. Future studies need to be conducted covering larger areas and population, and should be directed towards finding the exact cause for this high rate of thyroid autoimmunity.

## Conclusions

School children of eastern Nepal have adequate iodine nutrition. Thyroid dysfunction is sparse but thyroid autoimmunity is common in children. A strong association of thyroid autoimmunity with iodine nutrition and thyroid dysfunction was seen in this cohort.

## Data Availability

Data available from the authors on special request.

## Abbreviations

APDM: Ammonium persulphate digestion method
Free T3: Free triiodothyronine
Free T4: Free thyroxine
ID: Iodine deficiency
Tg: Thyroglobulin
TgAb: Antithyroglobulin antibody
TPO: Thyroid peroxidase
TSH: Thyroid stimulating hormone
UIC: Urinary iodine concentration
USI: Universal salt iodization
VDC: Village development commitee
WHO: World health organization
